# Crystal structure of 2,6-di­amino­pyridinium chloride

**DOI:** 10.1107/S2056989016002425

**Published:** 2016-02-13

**Authors:** Matthias Mastalir, Martina Schroffenegger, Berthold Stöger, Matthias Weil, Karl Kirchner

**Affiliations:** a Institute of Applied Synthetic Chemistry, TU Wien, Getreidemarkt 9/163, A-1060 Vienna, Austria; b Institute for Chemical Technologies and Analytics, Division of Structural Chemistry, TU Wien, Getreidemarkt 9/164-SC, A-1060 Vienna, Austria

**Keywords:** crystal structure, 2,6-di­amino­pyridinium cation, hybrid salt, hydrogen bonding

## Abstract

The asymmetric unit of the title salt, C_5_H_8_N_3_^+^·Cl^−^, comprises one half of the 2,6-di­amino­pyridinium cation (the other half being completed by the application of mirror symmetry) and one Cl^−^ counter-anion, also located on the mirror plane. The amino N atom shows a significant pyramidalization, with a dihedral angle of 30.4 (14)° between the least-squares planes of the amino group and the non-H atoms of the 2,6-di­amino­pyridinium moiety. In the crystal, the cationic mol­ecules and Cl^−^ counter-anions are arranged in sheets parallel to (001) consisting of alternating polar and non-polar parts associated with the the Cl^−^ anions, pyridinium and amino moieties, and the pyridine rings, respectively. N—H⋯Cl inter­actions within the polar part, as well as slipped π–π inter­actions in the non-polar part, help to establish the three-dimensional network.

## Abstract 2

The crystal structure of the organic–inorganic hybrid title salt is held together by N—H⋯Cl hydrogen bonds.

## Chemical context   

Pincer compounds are an important class of chelating ligands, and their metal complexes have attracted tremendous inter­est due to their high stability, activity, variability and applicability in organic synthesis and catalysis (Szabo & Wendt, 2014 ▸). Whereas a plethora of (mostly) precious transition-metal pincer complexes has been reported, information on group 6 pincer complexes is rather scarce. During a project aimed at the preparation and characterization of group 6 PNP pincer compounds (Öztopcu *et al.*, 2013 ▸; de Aguiar *et al.*, 2014 ▸; Mastalir *et al.*, 2016 ▸), crystals of the title salt, C_5_H_8_N_3_^+^·Cl^−^, were obtained accidentally through hydrolysis of the employed ligand *N,N*’-bis­(diiso­propyl­phosphino)-2,6-di­amino­pyridine in the presence of CrCl_3_·6H_2_O. Here we report on the crystal structure of this salt.
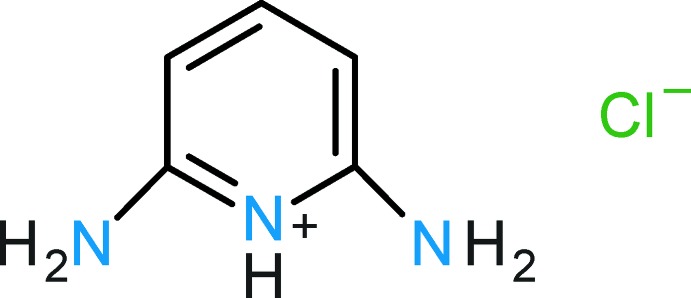


## Structural commentary   

**Figure 1:**
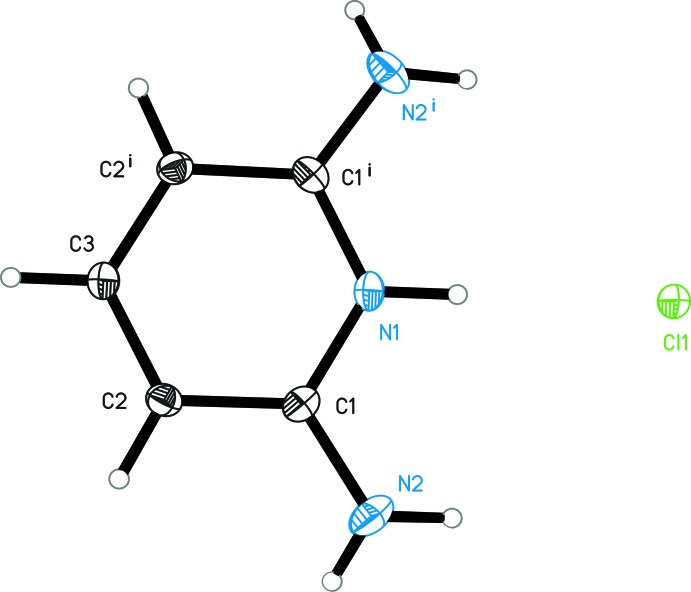
The mol­ecular structure of the cation and the inorganic anion in the title structure. Displacement ellipsoids are drawn at the 50% probability level. [Symmetry code: (i) *x*, −*y*, *z*.]

The cation of the title structure is protonated at the pyridine N atom (Fig. 1 ▸). The asymmetric unit comprises half a mol­ecule of the 2,6-di­amino­pyridinium cation, with a mirror plane running through the pyridinium group (N1—H1*N*1) and the *para*-C—H group (C3—H1*C*3); the Cl^−^ anion is also located on the mirror plane. In agreement with other 2,6-di­amino­pyridinium cations, the C—N(H)^+^—C angle involving the pyridinium group is enlarged [C1—N1—C1^i^ = 123.37 (8)°; symmetry code: (i) *x*, −*y*, *z*] whereas the angle between the pyridinium N atom and the C atom in the *ortho* position (bearing the amino group) and in the *meta* position is reduced [N1—C1—C2 = 118.83 (6)°]. This situation is reversed in 2,6-di­amino­pyridine due to the non-protonated ring N atom in this structure (Schwalbe *et al.*, 1987 ▸). A common feature of the non-protonated 2,6-di­amino­pyridine mol­ecule and the 2,6-di­amino­pyridinium cation is a significant pyramidalization of the amino N atom. In the title structure, the bond angle sum at this atom (N2) deviates with 349.0° clearly from the expected 360° for an ideal trigonal–planar group. The pyramidalization is also reflected by the dihedral angle of 30.4 (14)° between the least-squares planes of the amino group and the non-H atoms of the 2,6-di­amino­pyridinium moiety.

## Supra­molecular features   

The pyridinium N1—H1*N*1 group is the donor of a nearly linear hydrogen bond to the Cl^−^ counter anion (Table 1 ▸). The amino group also participates in the formation of N—H⋯Cl hydrogen bonds, albeit of explicit weaker nature. One hydrogen atom (H2*N*2) is clearly involved in hydrogen bonding with an H2*N*2⋯Cl1 distance of 2.63 Å and an N2—H2*N*2⋯Cl1 angle of 157°. Although the *D*⋯*A* contact involving the second hydrogen atom, H2*N*2, is 0.04 Å shorter than that of the other hydrogen bond of this group, the comparatively long H1*N*2⋯Cl distance of 2.88 Å and the very small N2—H1*N*2⋯Cl1 angle of 117° give room for inter­pretation whether or not this is a real hydrogen bond.

**Figure 2:**
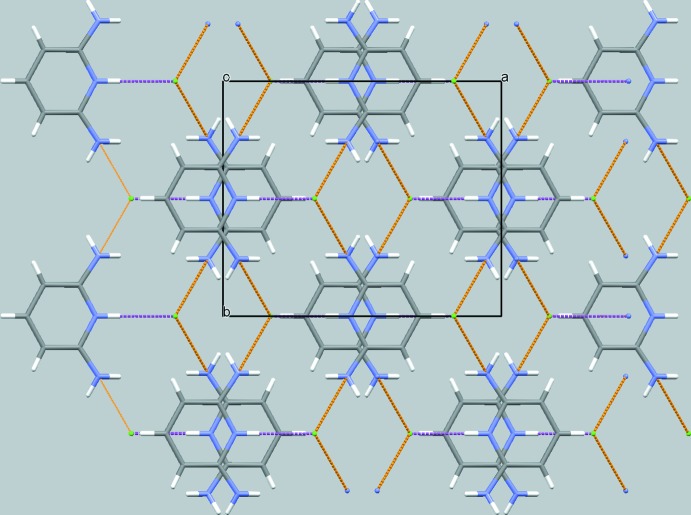
Crystal packing of the organic and inorganic components in the title structure in a projection along [001]. N—H⋯Cl hydrogen bonds involving the pyridinium group are shown as magenta dotted lines and those involving the amino group are shown as orange dotted lines.

In the crystal (Figs. 2 ▸ and 3 ▸), the cationic mol­ecules and anions are arranged into layers with alternating polar and non-polar parts extending parallel to (001). Adjacent polar parts, comprising the Cl^−^ anions and the pyridinium and amino moieties, are linked through N—H⋯Cl hydrogen bonds into sheets with a thickness of ≃ *c*/2. The non-polar parts, *i.e.* the pyridine rings, inter­act through slipped π–π stacking along [001] with a centroid-to-centroid distance of 3.5129 (6) Å; the corresponding plane-to-plane distance between the pyridine rings is 3.344 Å.

## Database survey   

A search in the CSD (Groom & Allen, 2014 ▸; CSD Version 5.31) revealed 87 different salts containing the 2,6-di­amino­pyridinium cation, with the majority of cases in the form of organic anions (46 representatives), followed by complex metal anions (31 representatives). Two structures are reported that contain additional metal cations and inorganic anions, and eight representatives are compiled with inorganic anions only, including the SiF_6_^2−^ salt (CSD code FOSXER; Gelmboldt *et al.*, 2009 ▸), the Br^−^ salt (GOLMIF; Turrell *et al.*, 2010 ▸), the BF_4_^−^ salt (IFOQAW; Benito-Garagorri *et al.*; 2007 ▸), the Br^−^ salt monohydrate (ILINEW; Haddad & Al-Far, 2003 ▸), the hydrogensulfate sulfate salt (KORRAM; Said & Naili, 2014 ▸), the ClO_4_^−^ salt (MIGWOP; Jazdoń *et al.*, 2007 ▸), the H_2_PO_4_^−^ salt (QEDHUE; Yu, 2012 ▸) and the NO_3_^−^ salt (XAKVAG; Kristiansson, 2000 ▸). It should be noted that the chemically most related anhydrous Br^−^ salt crystallizes in space group *I* 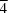
2*d* and hence shows no isotypism with the title Cl^−^ salt.

## Synthesis and crystallization   

*N,N*’-bis­(diiso­propyl­phosphino)-2,6-di­amino­pyridine (0.2 g, 0.53 mmol) was dissolved in dry tetra­hydro­furan (5 ml) under argon atmosphere. CrCl_3_·6H_2_O (0.134 g, 0.51 mmol) was added and the resulting mixture stirred for 4 h at room temperature. The formed purple solid was filtered off, washed with dry diethyl ether and dried. The solid was redissolved in aceto­nitrile for crystallization initiated by solvent diffusion with diethyl ether. The title compound grew in the form of yellow crystals as the only solid product. We assume that the Lewis acid CrCl_3_ in combination with water is able to cleave the P—N bond of the pincer compound accompanied by an *in situ* formation of HCl which eventually yields the title compound.

## Refinement   

All H atoms were clearly discernible from difference Fourier maps and were refined freely. Crystal data, data collection and structure refinement details are summarized in Table 2 ▸.

## Supplementary Material

Crystal structure: contains datablock(s) global, I. DOI: 10.1107/S2056989016002425/hb7564sup1.cif

Structure factors: contains datablock(s) I. DOI: 10.1107/S2056989016002425/hb7564Isup2.hkl

Supporting information file. DOI: 10.1107/S2056989016002425/hb7564Isup3.cml

CCDC reference: 1452262

Additional supporting information:  crystallographic information; 3D view; checkCIF report

## Figures and Tables

**Figure 3:**
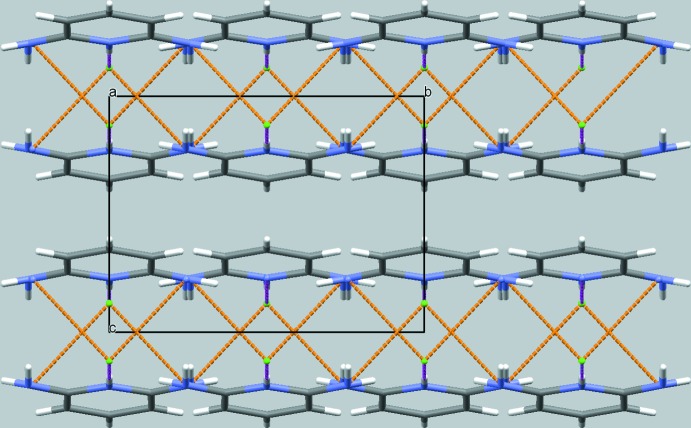
Crystal packing of the organic and inorganic components in the title structure in a projection along [100]. The colour code of the inter­molecular inter­actions is as in Fig. 2 ▸.

**Table 1:** Hydrogen-bond geometry (Å, °)

*D*—H⋯*A*	*D*—H	H⋯*A*	*D*⋯*A*	*D*—H⋯*A*
N1—H1*N*1⋯Cl1	0.90 (2)	2.18 (2)	3.0790 (11)	175.6 (19)
N2—H2*N*2⋯Cl1^i^	0.833 (13)	2.628 (13)	3.4086 (8)	156.5 (12)
N2—H1*N*2⋯Cl1^ii^	0.875 (13)	2.877 (13)	3.3601 (8)	116.8 (2)

Symmetry codes: (i) 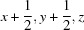; (ii) 
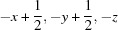
.

**Table 2:** Experimental details

Crystal data	
Chemical formula	C_5_H_8_N^3+^·Cl^−^
*M*_r_	145.6
Crystal system, space group	Monoclinic, *C*2/*m*
Temperature (K)	100
*a*, *b*, *c* (Å)	10.8046 (10), 9.0459 (9), 6.8108 (7)
β (°)	96.710 (2)
*V* (Å^3^)	661.11 (11)
*Z*	4
Radiation type	Mo *K*α
μ (mm^−1^)	0.48
Crystal size (mm)	0.52 × 0.38 × 0.23

Data collection	
Diffractometer	Bruker Kappa APEXII CCD
Absorption correction	Multi-scan (*SADABS*; Bruker, 2014 ▸)
*T*_min_, *T*_max_	0.80, 0.90
No. of measured, independent and observed [*I* > 3σ(*I*)] reflections	9529, 1538, 1407
*R*_int_	0.031
(sin θ/λ)_max_ (Å^−1^)	0.808

Refinement	
*R*[*F* ^2^ > 3σ(*F*)], *wR*(*F*), *S*	0.024, 0.038, 2.24
No. of reflections	1538
No. of parameters	64
H-atom treatment	All H-atom parameters refined
Δρ_max_, Δρ_min_ (e Å^−3^)	0.49, −0.18

Computer programs: *APEX2* (Bruker, 2014 ▸), *SAINT* (Bruker, 2014 ▸), *SUPERFLIP* (Palatinus & Chapuis, 2007 ▸), *JANA2006* (Petříček *et al.*, 2014 ▸), *XP* in *SHELXTL* (Sheldrick, 2008 ▸), *Mercury* (Macrae *et al.*, 2006 ▸) and *publCIF* (Westrip, 2010 ▸).

## supplementary crystallographic information

### Crystal data

**Table:**

C_5_H_8_N^3+^·Cl^−^	*F*(000) = 304
*M**_r_* = 145.6	*D*_x_ = 1.462 Mg m^−^^3^
Monoclinic, *C*2/*m*	Mo *K*α radiation, λ = 0.71073 Å
Hall symbol: -C 2y	Cell parameters from 6316 reflections
*a* = 10.8046 (10) Å	θ = 2.9–35.5°
*b* = 9.0459 (9) Å	µ = 0.48 mm^−^^1^
*c* = 6.8108 (7) Å	*T* = 100 K
β = 96.710 (2)°	Block, yellow
*V* = 661.11 (11) Å^3^	0.52 × 0.38 × 0.23 mm
*Z* = 4	

### Data collection

**Table:**

Bruker Kappa APEXII CCD diffractometer	1538 independent reflections
Radiation source: X-ray tube	1407 reflections with *I* > 3σ(*I*)
Graphite monochromator	*R*_int_ = 0.031
ω– and φ–scans	θ_max_ = 35.0°, θ_min_ = 3.0°
Absorption correction: multi-scan (*SADABS*; Bruker, 2014)	*h* = −17→17
*T*_min_ = 0.80, *T*_max_ = 0.90	*k* = −14→14
9529 measured reflections	*l* = −10→10

### Refinement

**Table:**

Refinement on *F*	0 constraints
*R*[*F*^2^ > 2σ(*F*^2^)] = 0.024	All H-atom parameters refined
*wR*(*F*^2^) = 0.038	Weighting scheme based on measured s.u.'s *w* = 1/(σ^2^(*F*) + 0.0001*F*^2^)
*S* = 2.24	(Δ/σ)_max_ = 0.030
1538 reflections	Δρ_max_ = 0.49 e Å^−^^3^
64 parameters	Δρ_min_ = −0.18 e Å^−^^3^
0 restraints	

### Fractional atomic coordinates and isotropic or equivalent isotropic displacement parameters (Å^2^)

**Table:**

	*x*	*y*	*z*	*U*_iso_*/*U*_eq_	
Cl1	0.17115 (2)	0	0.12206 (3)	0.01401 (7)	
N1	0.45600 (9)	0	0.22925 (12)	0.0132 (2)	
N2	0.44617 (7)	0.25682 (8)	0.22357 (10)	0.01951 (18)	
C1	0.51532 (7)	0.13257 (7)	0.25660 (9)	0.01272 (16)	
C2	0.64225 (7)	0.13396 (7)	0.32011 (10)	0.01334 (17)	
C3	0.70393 (10)	0	0.35111 (14)	0.0136 (2)	
H1C2	0.6796 (9)	0.2256 (14)	0.3421 (16)	0.019 (3)*	
H1C3	0.7905 (16)	0	0.392 (2)	0.015 (3)*	
H1N2	0.3742 (16)	0.2574 (17)	0.150 (2)	0.046 (4)*	
H2N2	0.4841 (12)	0.3350 (15)	0.2058 (19)	0.032 (3)*	
H1N1	0.3735 (19)	0	0.191 (3)	0.039 (5)*	

### Atomic displacement parameters (Å^2^)

**Table:**

	*U*^11^	*U*^22^	*U*^33^	*U*^12^	*U*^13^	*U*^23^
Cl1	0.01121 (13)	0.01270 (11)	0.01763 (12)	0	−0.00037 (8)	0
N1	0.0093 (4)	0.0176 (4)	0.0128 (3)	0	0.0018 (3)	0
N2	0.0192 (3)	0.0187 (3)	0.0214 (3)	0.0075 (2)	0.0056 (2)	0.0065 (2)
C1	0.0137 (3)	0.0143 (3)	0.0107 (2)	0.0025 (2)	0.0039 (2)	0.00180 (19)
C2	0.0136 (3)	0.0118 (3)	0.0149 (3)	−0.0012 (2)	0.0026 (2)	−0.00027 (19)
C3	0.0108 (4)	0.0154 (4)	0.0145 (4)	0	0.0011 (3)	0

### Geometric parameters (Å, º)

**Table:**

N1—C1	1.3622 (8)	C1—C2	1.3890 (10)
N1—C1	1.3622 (8)	C2—C3	1.3872 (9)
N1—H1N1	0.90 (2)	C2—H1C2	0.927 (12)
N2—C1	1.3538 (10)	C3—H1C3	0.944 (17)
N2—H1N2	0.875 (16)	C3—C2	1.3872 (9)
N2—H2N2	0.833 (13)		
			
C1—N1—C1	123.37 (8)	N2—C1—C2	123.35 (6)
C1—N1—H1N1	118.32 (4)	C1—C2—C3	118.60 (7)
C1—N1—H1N1	118.32 (4)	C1—C2—H1C2	117.0 (6)
C1—N2—H1N2	122.5 (10)	C3—C2—H1C2	124.4 (6)
C1—N2—H2N2	117.2 (9)	C2—C3—C2	121.75 (9)
H1N2—N2—H2N2	109.3 (13)	C2—C3—H1C3	119.12 (5)
N1—C1—N2	117.81 (7)	C2—C3—H1C3	119.12 (5)
N1—C1—C2	118.83 (6)		

### Hydrogen-bond geometry (Å, º)

**Table:**

*D*—H···*A*	*D*—H	H···*A*	*D*···*A*	*D*—H···*A*
N1—H1*N*1···Cl1	0.90 (2)	2.18 (2)	3.0790 (11)	175.6 (19)
N2—H2*N*2···Cl1^i^	0.833 (13)	2.628 (13)	3.4086 (8)	156.5 (12)
N2—H1*N*2···Cl1^ii^	0.875 (13)	2.877 (13)	3.3601 (8)	116.8 (2)

Symmetry codes: (i) *x*+1/2, *y*+1/2, *z*; (ii) −*x*+1/2, −*y*+1/2, −*z*.
